# Effect on capillary refill time of volume expansion and increase of the norepinephrine dose in patients with septic shock

**DOI:** 10.1186/s13054-023-04714-0

**Published:** 2023-11-06

**Authors:** Nicolas Fage, Francesca Moretto, Daniela Rosalba, Rui Shi, Christopher Lai, Jean-Louis Teboul, Xavier Monnet

**Affiliations:** 1 Service de Médecine Intensive-Réanimation, Hôpital de Bicêtre, DMU CORREVE, Inserm UMR S_999, FHU SEPSIS, Groupe de Recherche Clinique CARMAS, Université Paris-Saclay, AP-HP, Le Kremlin-Bicêtre, France; 2 Department of Medical Intensive Care, University Hospital of Angers, Angers, France; 3 MITOVASC Laboratory UMR INSERM (French National Institute of Health and Medical Research), 1083–CNRS 6015, University of Angers, Angers, France

**Keywords:** Capillary refill time, Hemodynamic, Norepinephrine, Microcirculation, Septic shock

## Abstract

**Background:**

Capillary refill time (CRT) has been suggested as a variable to follow during the course of septic shock. We systematically investigated the effects on CRT of volume expansion and norepinephrine.

**Methods:**

In 69 septic shock patients, we recorded mean arterial pressure (MAP), cardiac index (CI), and 5 consecutive CRT measurements (video method, standardized pressure applied on the fingertip) before and after a 500-mL saline infusion in 33 patients and before and after an increase of the norepinephrine dose in 36 different patients. Fluid responders were defined by an increase in CI ≥ 15%, and norepinephrine responders by an increase in MAP ≥ 15%.

**Results:**

The least significant change of CRT was 23%, so that changes in CRT were considered significant if larger than 23%. With volume expansion, CRT remained unchanged on average in patients with baseline CRT < 3 s (n = 7) and in all but one patient with baseline CRT ≥ 3 s in whom fluid increased CI < 15% (n = 13 “fluid non-responders”). In fluid responders with baseline CRT ≥ 3 s (n = 13), CRT decreased in 8 patients and remained unchanged in the others, exhibiting a dissociation between CI and CRT responses. The proportion of patients included > 24 h after starting norepinephrine was higher in patients with such a dissociation than in the other ones (60% vs. 0%, respectively). Norepinephrine did not change CRT significantly (except in one patient) if baseline CRT was ≥ 3 s and the increase in MAP < 15% (n = 6). In norepinephrine responders with prolonged baseline CRT (n = 11), it increased in 4 patients and remained unchanged in the other ones, which exhibited a dissociation between MAP and CRT responses.

**Conclusions:**

In septic shock patients with prolonged CRT, CRT very rarely improves with treatment when volume expansion increases cardiac output < 15% and increasing norepinephrine increases MAP < 15%. When the effects of fluid infusion on cardiac output and of norepinephrine on MAP are significant, the response of CRT is variable, as it decreases in some patients and remains stable in others which exhibit a dissociation between changes in macrohemodynamic variables and in CRT. In this regard, CRT behaves as a marker of microcirculation.

*Trial registration*: ClinicalTrial.gov (NCT04870892). Registered January15, 2021. Ethics committee approval CE SRLF 21-25.

**Supplementary Information:**

The online version contains supplementary material available at 10.1186/s13054-023-04714-0.

## Introduction

Among the clinical variables used to evaluate patients with acute circulatory failure at the bedside, the capillary refill time (CRT) is easily assessed [1–3]. A prolonged CRT has been found to be associated with high mortality rate in patients with septic [1, 4] or cardiogenic shock [5]. The interest in CRT was emphasized by the ANDROMEDA-SHOCK study, which demonstrated that, in patients with septic shock, resuscitation aimed at either normalizing CRT or normalizing or decreasing lactate levels during an 8-h intervention period had a similar effect on 28-day mortality [3, 6]. CRT is more and more considered as a core monitoring component during resuscitation of septic shock patients [7–10], particularly in low-resource settings [11, 12].

The aim of resuscitation in patient with septic shock is to restore organ perfusion. Thus, monitoring changes in CRT over time may be used to follow the perfusion status [3, 13]. However, the physiological determinants of CRT are not well defined. CRT reflects skin perfusion and vascular reactivity [14]. It should thus be influenced by the microcirculation structure and function, and by some macrocirculatory variables, such as mean arterial pressure (MAP) and cardiac output. A recent meta-analysis showed an inverse correlation between MAP and CRT [15]. Nevertheless, the effects on CRT of fluid infusion and norepinephrine, which are the two main treatments of septic shock, have not been systematically investigated.

Thus, the objective of this observational study performed in patients with septic shock, was to describe the changes of CRT during volume expansion and an increase in the norepinephrine dose and their macrocirculatory determinants.

## Methods

This prospective study was conducted between January 18, 2021, and October 13, 2022 in the 25-bed intensive care unit (ICU) of a tertiary teaching hospital. This study was approved by the ethics committee of the French Intensive Care Society (CE SRLF 21–25) and registered on ClinicalTrials.gov (NCT04870892). It was conducted according to the STROBE guidelines [16] (Additional File 1: Appendix 1). At the time of inclusion, inform consent was obtained from patients or their next of kin. All patients and/or relatives agreed to participate.

### Patients

Inclusion criteria were (i) age ≥ 18 years old, (ii) presence of septic shock according to the current definition [17], (iii) monitoring already in place by a transpulmonary thermodilution device with calibrated pulse contour analysis (PiCCO2, Pulsion Medical Systems, Getinge, Feldkirchen, Germany) and (iv) volume expansion or increase of the norepinephrine dose, as required by the attending physicians. Exclusion criteria were (i) extracorporeal membrane oxygenation, (ii) pregnancy, (iii) dark skin (skin phototypes V or VI according to Fitzpatrick classification [18]), (iv) past medical history of Raynaud phenomenon, (v) in patients of the “volume expansion group”, necessity of changing the dose of any vasoactive drug during the time of investigation and, in the norepinephrine group, necessity to infuse fluid or change any vasoactive drug during the time of investigation and (vi) in the “norepinephrine group”, impossibility to obtain a stabilization of the mean arterial pressure (MAP) within one hour after the change in the dose of norepinephrine. Non-inclusion criteria were (i) unavailability of investigators and (ii) refusal to participate in this study. The decision to perform volume expansion was typically taken in the presence of decreased urine output and/or increased lactate or carbon dioxide partial pressure-derived indices and/or mottling or increased CRT. The decision to increase norepinephrine was taken according to the targeted MAP, which typically took into account previous hypertension and/or previous chronic kidney disease and/or elevated intravascular pressure and intra-abdominal pressure.

### Capillary refill time measurements

CRT measurement was recorded at the palmar surface of the right index using a standardized method which has been previously described [19]. In order to measure changes of CRT of small amplitude, we recorded it with a smartphone’s video camera (Samsung Galaxy A7, 16 megapixels). The lightning conditions were controlled by using the flashlight system of the smartphone. Pressure on the finger was applied through the piston of a 10-mL syringe (PosiFlush XS, Becton Dickinson, Franklin lakes, NJ, USA). The syringe was filled with 10 mL of air and its port was occluded. The pressure applied by the piston on the fingertip was standardized by compressing the air volume in the syringe from 10 to 7 mL during 7 s.

Five CRT measurements were made at each hemodynamic condition in less than 3 min and the videos were analyzed a posteriori by one reader using the freeware Kinovea (www.kinovea.org). The reader of the videos was blinded to the clinical and hemodynamic conditions of the patient.

### Hemodynamic measurements

Patients were equipped with a thermistor-tipped arterial femoral catheter and an internal jugular vein catheter, as required by the PiCCO2 device [20]. Pressure sensors were fixed on the upper arm and referenced to the right atrium, corresponding to the axillary line and zeroing was performed against atmospheric pressure [21]. CI was measured with transpulmonary thermodilution, using the PiCCO2 device performed by injecting three 15-mL cold boluses of normal saline through the central venous catheter and averaging the result obtained from three consecutive injections [22]. CVP was measured at end expiration. Blood temperature was monitored through the thermistor of the PiCCO2 device. Fluid responsiveness was defined by a fluid-induced increase in CI ≥ 15% [20].

### Study design

For all patients, CRT, arterial lactate, hemodynamic variables (systemic arterial pressure, central venous pressure (CVP), heart rate, cardiac index (CI)), ventilatory settings, norepinephrine dose and core temperature were assessed at baseline.

In patients of the “volume expansion group”, a 500-mL fluid bolus of NaCl 0.9% was infused intravenously over 15 min. In patients of the “norepinephrine group”, the norepinephrine dose was increased as decided by the clinicians in charge of the patient. The same variables as before intervention were measured after, immediately after the end of fluid infusion in the “volume expansion group”, and after stabilization of MAP (i.e., variation < 15% of MAP) in the “norepinephrine group”, even if the MAP level achieved was higher than the initial MAP target. Arterial lactate was measured only before interventions.

### Statistical analysis

Quantitative variables, presented as median [interquartile range], were compared with the Mann–Whitney U test between groups of patients and with Wilcoxon matched-pairs test between study times. Qualitative variables, presented as the absolute value [percentage], were compared with Fisher’s exact test.

In each patient, we calculated the coefficient of variation (CV) of the CRT as being the standard deviation of the five measurements performed at baseline, divided by the number of measurements. The coefficient of error (CE) was obtained by using the formula CE = CV/√n, where n was the number of measurements. The precision was calculated as being two CE for averaged measurements. The least significant change (LSC), which is the minimum change that needs to be measured by a device in order to recognize a real change [23], was calculated using the following equation: LSC = CE × 1.96 × √2.

Regression linear analysis was used to analyze the determinants of the CRT and of its changes. In the first step, univariate linear regression analysis was conducted separately for each variable of interest. In the second step, a backward multivariate linear regression analysis was built using variables with p-value < 0.1 in univariate linear regression.

Based on a previous study evaluating the effect of volume expansion on CRT [19], considering an α-risk of 5% and a ß-risk at 20%, predicting a CRT change of 0.3 ± 0.5 s. during volume expansion and an identical change during the increase in norepinephrine dose, we estimated that 32 patients with volume expansion and 32 patients with an increase in norepinephrine dose should be included in the study.

Statistical analysis was performed using Prism GraphPad Software v10.0.0 (San Diego, CA, USA). All tests were two-sided, and p-values below 0.05 were considered as statistically significant.

## Results

### Patient characteristics

The baseline characteristics of the 69 included patients (33 in the volume expansion group and 36 in the norepinephrine group) are summarized in Table 1. Patients were included in the study 24 (10–76) hours after the introduction of norepinephrine (20 (6–33) hours in the “volume expansion” group and 58 (15–197) hours in the “norepinephrine” group). The SAPSII and SOFA scores were lower in the “norepinephrine” group than in the “volume expansion” group, while other variables were similar (Table 1**)**.

**Table 1:** Patient characteristics at inclusion and ICU mortality

	All patients n = 69	Volume expansion n = 33	Increase of norepinephrine dose n = 36	p value
Age – years	66 [56–74]	67 [55–78]	66 [59–74]	0.71
Male sex – no (%)	40 (58)	16 (48)	24 (66)	0.15
BMI – kg/m^2^	27 [22–32]	25 [20–32]	27 [25–33]	0.17
SAPS II	52 [46–58]	54 [49–61]	48 [38–57]	0.02
SOFA	9 [8–11]	10 [8–13]	8 [7–11]	0.02
Arterial lactate – mmol/L	2.7 [2.0–4.4]	3.8 [2.3–4.8]	2.2 [1.8–3.1]	0.001
Pre-existing condition – no (%)				
Ischemic heart disease	10 (14)	5 (15)	5 (14)	0.99
Chronic heart failure	6 (9)	3 (9)	3 (9)	0.99
COPD	1 (1)	0 (0)	1 (3)	0.99
Chronic kidney disease	6 (9)	4 (12)	2 (6)	0.41
Diabetes mellitus	23 (33)	9 (27)	14 (41)	0.44
Cirrhosis	6 (9)	5 (15)	1 (3)	0.10
Hypertension	36 (52)	16 (48)	20 (56)	0.63
Cancer or autoimmune disease	18 (26)	7 (21)	11 (31)	0.42
Source of infection – no (%)				
Pneumonia	43 (62)	22 (67)	21 (58)	0.62
Abdominal infection	10 (14)	5 (15)	5 (14)	0.99
Urinary tract infection	3 (4)	1 (3)	2 (5)	0.99
Endocarditis	3 (4)	3 (9)	0 (0)	0.10
Catheter-related infection	1 (1)	0 (0)	1 (3)	0.99
Soft tissues infection	1 (1)	1 (3)	0 (0)	0.48
Undetermined etiology	8 (11)	1 (3)	7 (19)	0.06
Mechanical ventilation	60 (87)	27 (82)	33 (91)	
ICU mortality – no (%)	28 (41)	15 (45)	13 (36)	0.47

Values are presented as number (%) or median (interquartile range). *P* value refers to the difference between the groups “volume expansion” and “increase of norepinephrine dose”

*BMI* Body mass index *C*_*RS*_ Compliance of the respiratory system, *COPD* Chronic obstructive pulmonary disease, *PEEP* Positive end-expiratory pressure, *SOFA* Sequential organ failure assessment, *SAPS* Simplified acute physiology score

### Precision of the CRT measurements

The LSC of CRT was 22.4 (14.8–28.0)%, 24.5 (16.6–31.2)% and 26.6 (14.8–36.8)% if 5, 4 and 3 measurements of CRT were averaged, respectively (Additional File 1: Figure S1). Therefore, for the rest of the study, a change in CRT ≥ 23% obtained from 5 averaged measurements was considered as significant.

### Effects of volume expansion

These effects are reported in Table 2 and 3, and in Additional File 1: Tables S1 and S2. Considering all patients together, volume expansion significantly decreased heart rate and increased MAP, diastolic arterial pressure, CI, CVP and CRT (Table 2). A fluid-induced increase in CI ≥ 15% was observed in 17 patients (51%). In these fluid responders, MAP significantly increased by 30 (10–48)% and CI by 25 (19–41)% with fluid infusion (Table 3).

**Table 2:** Effects of volume expansion (Upper Panel) and of the increase in norepinephrine (Lower Panel) on hemodynamic variables

	Before intervention	After invention	*p*-value
*Volume expansion*			
MAP–mmHg	70 [62–79]	82 [75–101]	< 0.0001
DAP–mmHg	53 [47–62]	62 [56–73]	0.003
CVP–mmHg	7 [4–9]	10 [7–12]	< 0.0001
CI–L/min/m^2^	2.57 [2.06–3.01]	2.91 [2.33–3.54]	0.0001
HR–beats/min	106 [86–118]	103 [81–115]	0.02
Blood temperature–°C	36.9 [36.0–37.6]	36.8 [35.7–37.3]	< 0.0001
Arterial lactate–mmol/L	3.8 [2.3–4.8]	–	
Norepinephrine			
Rate–µg/kg/min	0.74 [0.28–1.67]	–	
Delay between initiation and CRT–hour	21 [8–33]	–	
CRT–sec	5.18 [3.24–7.44]	4.18 [2.77–5.94]	< 0.0001
*Increase in the norepinephrine dose*			
MAP–mmHg	66 [60–75]	85 [80–103]	< 0.0001
DAP–mmHg	50 [45–60]	62 [58–76]	< 0.0001
CVP–mmHg	9 [7–13]	12 [8–14]	0.048
CI–L/min/m^2^	2.88 [2.00–3.64]	2.94 [2.38–3.95]	< 0.0001
HR–beats/min	87 [73–103]	87 [72–98]	0.59
Blood temperature–°C	37.0 [36.1–37.4]	36.9 [36.1–37.3]	0.62
Lactate–mmol/L	2.2 [1.8–3.1]	–	
Norepinephrine			
Rate–µg/kg/min	0.23 [0.08–0.51]	0.42 [0.30–0.74]	< 0.0001
Delay between initiation and CRT–hour	58 [15–197]	–	
CRT–s	2.73 [1.63–4.88]	2.34 [1.30–4.10]	0.008

Values are presented as number (%) or median (interquartile range). *p*-values refer to the difference between before and after the intervention

*CI* Cardiac index, *C*_*RS*_ Compliance of the respiratory system, *CRT* Capillary refill time, *CVP* Central venous pressure, *DAP* Diastolic arterial pressure, *HR* Heart rate, *MAP* Mean arterial pressure

**Table 3:** Effects of volume expansion on hemodynamic variables in fluid responders (upper panel) and non-responders (lower panel)

	Before VE	After VE	*p*-value
*Fluid responders n = 17*			
MAP–mmHg	68 [61–77]	83 [78–104]	< 0.0001
DAP–mmHg	53 [47–58]	64 [56–70]	0.0002
CVP–mmHg	6 [4–9]	9 [5–12]	0.002
CI–L/min/m^2^	2.34 [1.69–3.06]	3.17 [2.28–4.03]	< 0.0001
HR–beats/min	112 [72–116]	107 [76–116]	0.40
Blood temperature–°C	37.0 [36.0–37.7]	36.8 [35.7–37.4]	< 0.0001
Arterial lactate–mmol/L	2.8 [2.0–4.6]	–	–
Norepinephrine			
Rate—µg/kg/min	0.56 [0.28–1.42]	0.56 [0.28–1.42]	–
Delay between initiation and CRT—hour	18 [4–25]	–	
CRT–s	5.49 [3.02–7.57]	3.41 [2.35–4.78]	< 0.0001
*Fluid non-responders n = 16*			
MAP–mmHg	72 [62–72]	80 [70–98]	0.005
DAP–mmHg	54 [48–65]	61 [52–73]	0.01
CVP–mmHg	8 [6–10]	10 [8–15]	0.002
CI–L/min/m^2^	2.71 [2.24–2.99]	2.73 [2.31–3.03]	0.73
HR–beats/min	104 [91–122]	100 [84–114]	0.006
Blood temperature–°C	36.8 [35.5–37.2]	36.7 [35.3–37.0]	0.001
Lactate–mmol/L	4.6 [2.4–5.7]	–	–
Norepinephrine			
Rate–µg/kg/min	0.79 [0.47–1.79]	0.79 [0.47–1.79]	–
Delay between initiation and CRT–hour	22 [11–39]	–	–
CRT–s	4.82 [3.16–7.45]	4.43 [3.01–7.44]	0.09

Values are presented as median [interquartile range]. *p*-value refer to the difference between before and after volume expansion

*CI* Cardiac index, *CRT* Capillary refill time, *CVP* Central venous pressure, *DAP* Diastolic arterial pressure, *HR* Heart rate, *MAP* Mean arterial pressure, *VE* Volume expansion

**Fig. 1:**
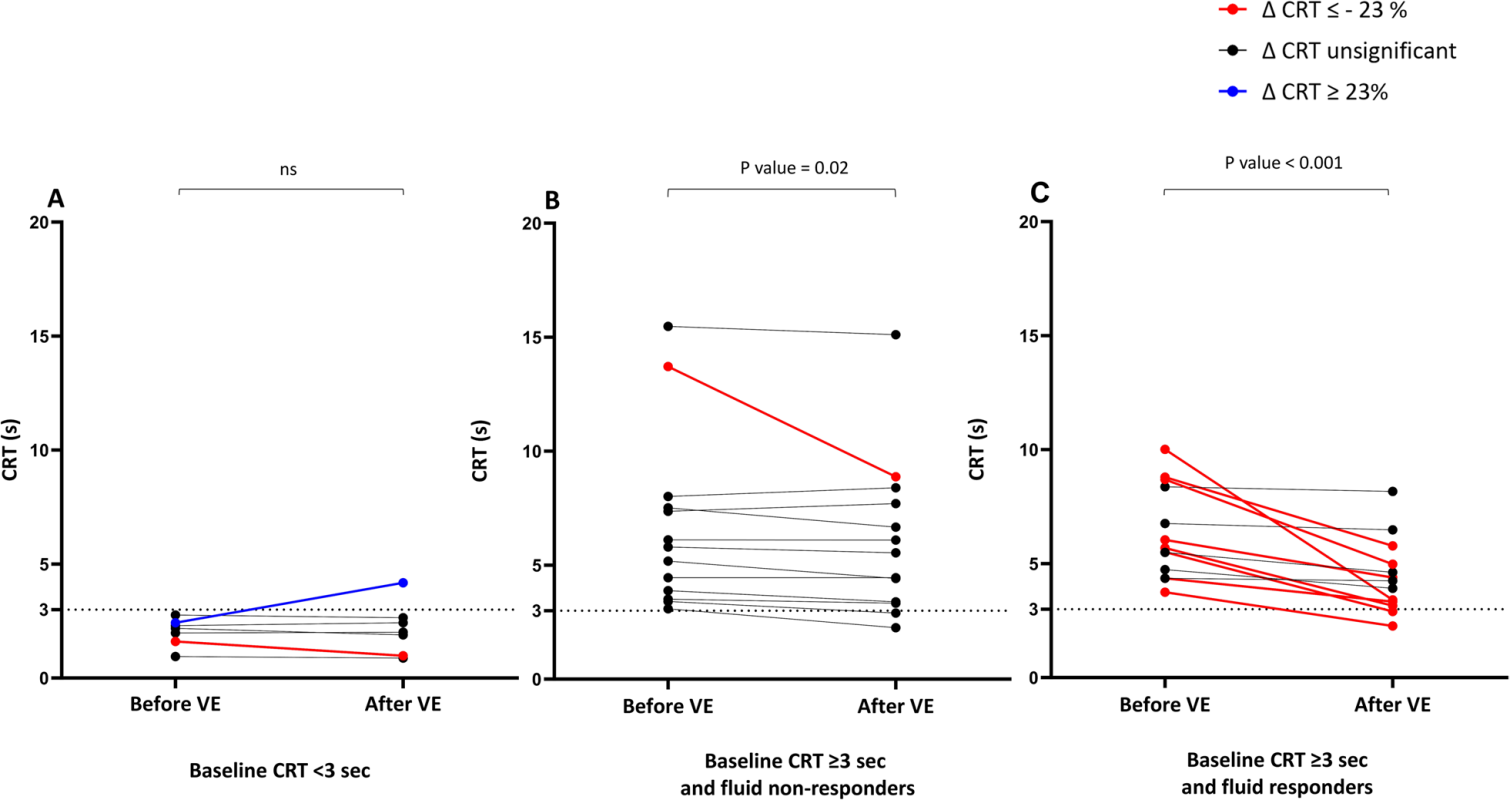
Evolution of the capillary refill time during volume expansion in patients with baseline CRT < 3 s (A, n = 7), patients with baseline CRT ≥ 3 s and fluid non-responders (B, n = 13) and patients with baseline CRT ≥ 3 s and fluid responders (C, n = 13)

On average, CRT remained unchanged with fluid infusion in patients with a baseline CRT < 3 s (Fig. 1A**,** Additional File 1: Table S1). In all fluid non-responders with baseline CRT ≥ 3 s but one, CRT remained unchanged (Fig. 1B**,** Additional File 1: Table S1). In the only fluid non-responder with a baseline CRT ≥ 3 s in whom CRT decreased significantly, it decreased from 13.7 to 8.9 s, while CI changed from 3.75 to 3.76 L/min/m^2^ and MAP from 82 to 86 mmHg.

In fluid responders with a baseline CRT ≥ 3 s, CRT decreased on average (Fig. 1C). It decreased ≥ 23% in 8 patients and remained unchanged in 5 patients. The time between the onset of septic shock and fluid infusion did not differ between these groups (10 [1.96–19.24] hours vs. 24 [3.6–31.3] hours, respectively, *p* = 0.28) (Additional File 1: Table S2).

The proportion of patients included > 24 h after starting norepinephrine was higher in patients whose CRT remained unchanged (n = 3, 60%) than patients with decreased their CRT (n = 0, 0%, *p* value = 0.03).

### Effects of increasing the dose of norepinephrine

In the 36 patients of this group, norepinephrine was increased from 0.23 [0.08–0.51] to 0.42 [0.30–0.74] µg/kg/min (Table 2). Considering all patients together, increasing norepinephrine induced a significant increase in MAP, diastolic arterial pressure, CI, CVP and CRT (Table 2). A norepinephrine-induced increase in MAP ≥ 15% was observed in 28 patients (78%). In these patients, MAP significantly increased by 30 (20–47)% and CI by 12 (4–20)% with the increase in norepinephrine dose (Additional File 1: Table S3).

**Fig. 2:**
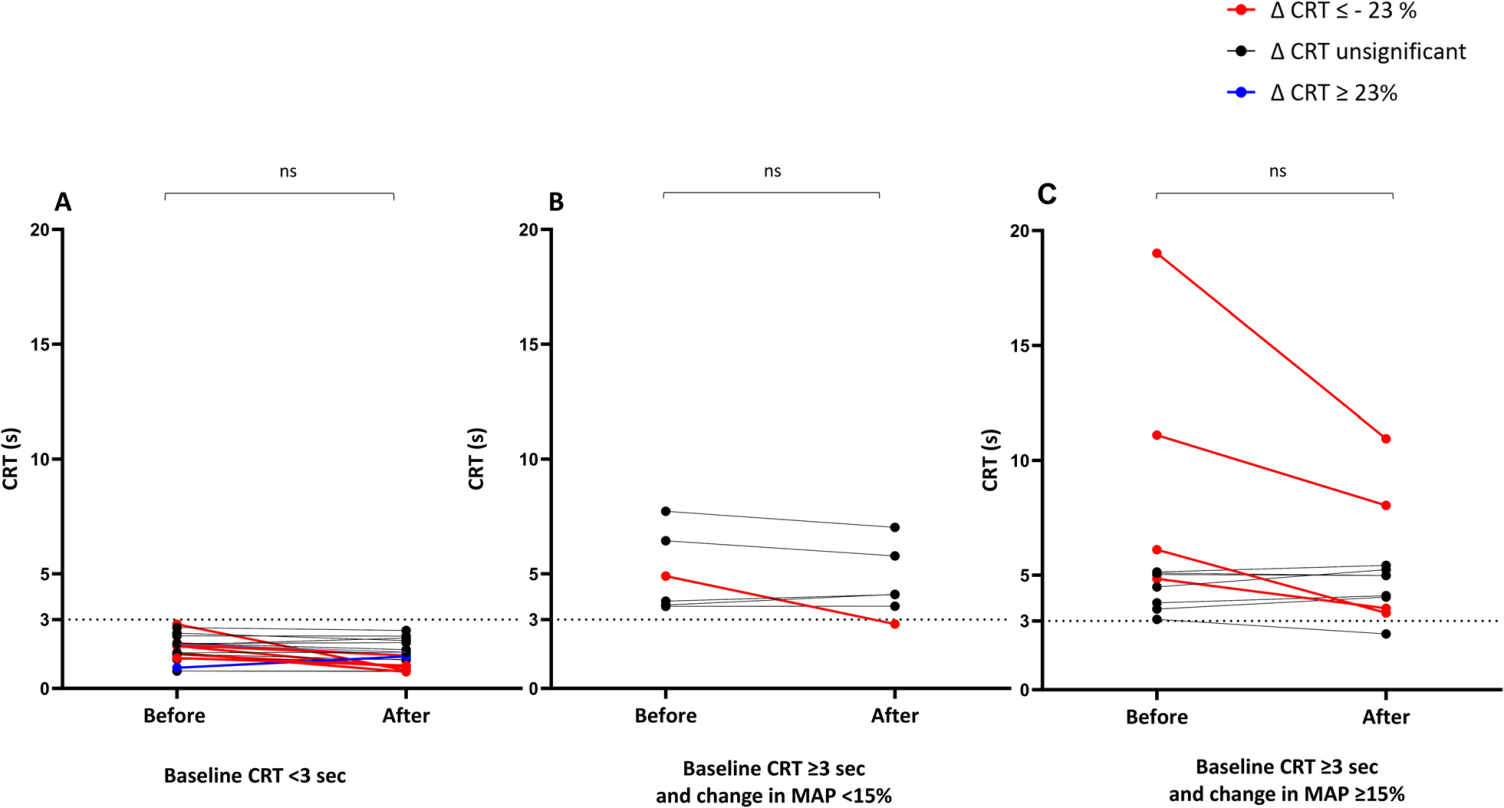
Evolution of the capillary refill time during norepinephrine increase in patients with baseline CRT < 3 s (A, n = 19), baseline CRT ≥ 3 s and change in MAP < 15% (B, n = 6) and baseline CRT ≥ 3 s and increase in MAP ≥ 15% (C, n = 11)

On average, CRT remained unchanged with the increase in norepinephrine in patients with a baseline CRT < 3 s (Fig. 2A**,** Additional File 1: Table S4). In these patients, it decreased ≥ 23% in 5 cases, increased > 23% in one case, and remained unchanged in the remaining cases. In all but one patient with baseline CRT ≥ 3 s and in whom norepinephrine increased MAP < 15%, CRT remained unchanged. In the only patient with baseline CRT ≥ 3 s with MAP increase < 15% in whom CRT decreased significantly, it decreased from 4.9 to 2.8 s, while CI changed from 2.65 to 2.70 L/min/m2 and MAP from 73 to 83 mmHg.

In patients with baseline CRT ≥ 3 s and in whom increasing norepinephrine increased MAP ≥ 15%, CRT decreased on average (Fig. 2C**,** Additional File 1: Table S4). It decreased by ≥ 23% in 4 patients and remained unchanged in 7 patients. The time between the onset of septic shock and fluid infusion did not differ between these groups (7 [3–411], hours vs. 171 [24–259] hours, respectively, *p* = 0.23). CRT decreased in 1 of the 5 patients in whom norepinephrine changed MAP < 15% (20%) included after 24 h from the onset of septic shock, and in 5 of the 11 patients in whom norepinephrine changed MAP ≥ 15% (45%) included within 24 h from the onset of septic shock (*p* = 0.59).

### Determinants of the absolute value of CRT

At univariate regression analysis, the absolute value of CRT was correlated with lactate, heart rate, norepinephrine dose, sex, SAPS II and ICU mortality (Additional File 1: Table S5). The correlation between CRT on the one hand, and CI, MAP and CVP on the other hand is shown in Additional File 1: Figure S2). At multivariate analysis including all physiological variables of interest with a *p* value < 0.1 in univariate linear regression analysis, only lactate at inclusion was significantly associated with the absolute value of CRT (Additional File 1: Table S6).

## Discussion

This study conducted in patients with septic shock showed that fluid infusion has very little chance to decrease CRT significantly if its baseline value is < 3 s and if baseline CRT is ≥ 3 s but the patient is fluid unresponsive, while in fluid responders with a baseline CRT ≥ 3 s, CRT decreases in some patients and remains unchanged in other ones. Increases in norepinephrine may reduce CRT in patients with baseline value < 3 s. In patients with baseline CRT ≥ 3 s, increasing norepinephrine has little chance to decrease CRT if the MAP change is < 15%, while the decrease of CRT is inconstant in patients with baseline CRT ≥ 3 s in whom norepinephrine increases MAP ≥ 15%.

In critically ill patients, CRT is considered as a valuable tool to assess tissue perfusion [13]. As such, it should be useful to evaluate the hemodynamic status in addition to macrohemodynamic variables such as heart rate, MAP, CVP and cardiac output [24]. The ANDROMEDA-SHOCK study conducted in septic shock patients showed that a CRT-targeted resuscitation was not different from lactate-targeted resuscitation regarding 28-day mortality in septic shock patients, but led to less organ dysfunctions at 72h and lower mortality in the subgroup of patients with less organ dysfunctions at baseline [3]. This study suggests that changes in CRT should be monitored to follow tissue perfusion during the course of septic shock. Our study aimed at systematically describing these CRT changes induced by the two main hemodynamic treatments of septic shock, *i.e.*, volume expansion and norepinephrine.

Regarding fluid infusion, the first important result was that CRT remained unchanged in all but one patient with baseline CRT < 3 s. This confirms that CRT reflects tissue perfusion, which should remain unchanged with fluids if normal. In fluid non-responders, CRT remained unchanged in all patients but one. This suggests that no significant improvement in tissue perfusion can be expected from a fluid bolus if cardiac output does not increase significantly. One hypothesis explaining the exception of the only fluid non-responder in whom CRT improved with fluid could be the positive rheologic effect of volume expansion [25], but we could not investigate this. In fluid responders with a prolonged CRT at baseline, the response of CRT was variable, shortening in some patients and remaining unchanged on some others. This suggests that in some cases, no improvement in tissue perfusion results from a significant increase in cardiac output, which could be explained by the dissociation between macro and microcirculatory variables that has been largely acknowledged in septic shock patients [26]. In our study, patients exhibiting such a dissociation between responses in cardiac output and in CRT tended to be included later than the other ones, but the difference was not obvious, which is in line with the fact that microcirculatory dysfunction is delayed during septic shock. Our results support a persistence of the interaction between the macro and microcirculation at the early phase of the septic shock [26].

The response of CRT to norepinephrine infusion was overall similar to the response to the fluid bolus: no change on average in patients with baseline CRT < 3 s, almost no patient with a decrease in CRT when baseline CRT was ≥ 3 s and the increase in MAP was small, variable response of CRT to norepinephrine increase in patients with altered CRT at baseline and change in MAP ≥ 15%. This response of CRT to norepinephrine has not been investigated, as far as we know. First, among all patients in whom the dose of norepinephrine was increased, CRT increased in one patient only, suggesting that the vasoconstrictive effect of the drug does not affect CRT in most cases. Second, as with fluid infusion, no improvement of CRT could be expected in patients with long baseline CRT if MAP increased to a small extent. As with fluid again, when baseline CRT was ≥ 3 s and MAP increased ≥ 15%, the CRT response was variable, exhibiting a dissociation between MAP and CRT changes in some patients. Again, If one agrees that CRT is at least partially determined by properties of the microcirculation [27], this is in accordance with studies reporting a large variability in microvascular responses to norepinephrine changes [28]. This is also consistent with a previous study showing that increasing the level of MAP target was not linked with the change in mottling, another marker of skin perfusion [29]. However, this interpretation must be cautious. First, few patients were observed in each subgroup. Second, MAP changes were classified around the arbitrary 15% threshold. Third, it is impossible to assess the precise factor that explained the behavior of changes CRT during norepinephrine change, and it may be possible that the effect of the MAP increase was counterbalanced by the concomitant vasoconstriction.

We attempted to determine the factors explaining CRT absolute value through univariate and multivariate analyses. Our observation of an association between arterial lactate level and CRT was consistent with the literature [1]. Even though CRT is related to skin blood flow [14, 30], we found no independent association between CRT absolute value on the one side and MAP and CVP values, corroborating the fact that it has other determinants than the macrocirculatory variables.

For measuring CRT, we chose a standardized method that has been previously described [19] rather than the method used in clinical practice, as for instance in the ANDROMEDA-SHOCK study [3]. The reason was that we needed a precise measurement of CRT, allowing us to evaluate the precision of the variable and determining which changes should be considered as significant and non-significant. Interestingly, we found exactly the same value of CRT precision when this method is used. However, we acknowledge that this complex method is not made for clinical use.

Our study may have significant clinical applications. First, the fact that no CRT improvement could be expected from volume expansion when it is normal at baseline strongly suggests that as when lactate, venous oxygen saturation, carbon dioxide-derived indices are normal, fluid bolus infusion should not be considered. Second, the absence of improvement in CRT in almost all fluid non-responders even when CRT was prolongated at baseline is another argument for avoiding fluid infusion in the absence of preload responsiveness, and for assessing that responsiveness. Of note, Jacquet-Lagrèze et al. nicely showed that the response of CRT to a fluid bolus is well predicted by the CRT changes induced by passive leg raising [19], suggesting that when preload responsiveness is tested, it should be assessed not only on cardiac index or surrogates [31], but also on CRT. Third, the variable response of CRT in fluid responders with altered CRT must be considered in line with the variable response of lactate or tissue oxygenation that has been already demonstrated. Finally, the dissociation between the response of MAP and of CRT when norepinephrine in increase in patients with prolongated CRT at baseline suggests that, as during fluid resuscitation, it should be carefully measured when assessing the effects of the drug changes.

Finally, our study has several limitations. First, defining different subgroups within our population reduced the number of patients in each. Second, we did not investigate microcirculation nor skin blood flow, which may have allowed us to better investigate all potential determinants of CRT. Third, we did not assess the effects of fluid infusion and of changes in norepinephrine dose on tissue oxygenation variables. Fourth, we did not include patients at the very early phase of septic shock, so that MAP was restored in most of our patients at baseline, and our results may have been different in patients without any prior resuscitation.

## Conclusion

In patients with septic shock with prolongated CRT, CRT very rarely improves with treatment when volume expansion increases cardiac output < 15% and increasing the norepinephrine dose increases MAP < 15%. When the effects of fluid infusion on cardiac output and of norepinephrine on MAP are significant, the response of CRT is dissociated, as it decreases in some patients and remains stable in others. In this regard, CRT behaves as a marker of microcirculation.

### Supplementary Information

**Additional file 1.** Supplementary information on further results.

## Data Availability

The datasets used and/or analyzed during the current study are available from the corresponding author on reasonable request.

## Abbreviations

CI: Cardiac index
CRT: Capillary refill time
CVP: Central venous pressure
ICU: Intensive care unit
MAP: Mean arterial pressure
