# Supplementary material for: Effect on capillary refill time of volume expansion and increase of the norepinephrine dose in patients with septic shock
Source: Crit Care. 2023 Nov 6;27:429. doi: 10.1186/s13054-023-04714-0 (PMC10629142; doi:10.1186/s13054-023-04714-0)
Supplement: Supplementary file 1 — Additional file 1. Supplementary information on further results. [file 13054_2023_4714_MOESM1_ESM.docx]

**Effect on capillary refill time of volume expansion and increase of norepinephrine dose in patients with septic shock.**

*Nicolas FAGE^1,2,3^, Francesca MORETTO^1^, Daniela ROSALBA^1^, Rui SHI^1^, Christopher LAI^1^, Jean-Louis TEBOUL^1^, Xavier MONNET^1^.*

^1^Université Paris-Saclay, AP-HP, Service de médecine intensive-réanimation, Hôpital de Bicêtre, DMU CORREVE, Inserm UMR S_999, FHU SEPSIS, Groupe de recherche clinique CARMAS, Le Kremlin-Bicêtre, France.

^2^Department of Medical Intensive Care, University Hospital of Angers, Angers, France.

^3^MITOVASC Laboratory UMR INSERM (French National Institute of Health and Medical Research), 1083 – CNRS 6015, University of Angers, Angers, France.

**Online supplementary material**

# **SUPPLEMENTARY TABLES**

## **Supplementary table 1. Effect of volume expansion on hemodynamic variables in baseline CRT <3 seconds patients (upper panel), baseline CRT ≥3seconds and fluid unresponsive patients (middle panel) and responsive patients (lower panel).**

| **Baseline CRT <3 seconds**  **n = 7** | | | |
| --- | --- | --- | --- |
|  | **Before VE** | **After VE** | **p-value** |
| MAP – mmHg | 70 [59 – 76] | 77 [57 – 81] | 0.08 |
| DAP – mmHg | 52 [48 – 63] | 62 [44 – 67] | 0.31 |
| CVP – mmHg | 8 [4 – 15] | 10 [5 – 17] | 0.06 |
| CI – L/min/m² | 2.19 [1.70 – 3.03] | 2.44 [2.01 – 3.54] | 0.11 |
| HR – beats/min | 110 [68 – 116] | 82 [66 – 104] | **0.03** |
| Arterial lactate – mmol/L | 2.7 [2.3 – 4.5] | - | - |
| Norepinephrine |  |  |  |
| rate – µg/kg/min | 0.51 [0.12 – 1.11] | - | - |
| delay between initiation and CRT – hour | 35 [24 – 42] | - | - |
| CRT – s. | 2.18 [1.60 – 2.42] | 2.01 [0.98 – 2.65] | 0.81 |
| **Baseline CRT ≥3 seconds and fluid unresponsiveness**  **n = 13** | | | |
|  | **Before VE** | **After VE** | **p-value** |
| MAP – mmHg | 75 [66 – 82] | 86 [75 – 100] | **0.004** |
| DAP – mmHg | 55 [48 – 66] | 62 [55 – 76] | **0.006** |
| CVP – mmHg | 8 [6 – 14] | 10 [8 – 16] | **0.005** |
| CI – L/min/m² | 2.92 [2.43 – 3.20] | 2.91 [2.33 – 3.11] | 0.73 |
| HR – beats/min | 103 [94 – 122] | 103 [90 – 119] | **0.01** |
| Lactate – mmol/L | 4.7 [3.1 – 9.6] | - | - |
| Norepinephrine |  |  |  |
| rate – µg/kg/min | 0.83 [0.56 – 1.90] | - | - |
| delay between initiation and CRT – hour | 21 [9 – 34] | - | - |
| CRT – s. | 5.79 [3.70 – 7.77] | 5.55 [3.63 – 8.05] | **0.02** |
| **Baseline CRT ≥3 seconds and fluid responsiveness**  **n = 13** | | | |
|  | **Before VE** | **After VE** | **p-value** |
| MAP – mmHg | 68 [61 – 75] | 85 [77 – 107] | **0.007** |
| DAP – mmHg | 53 [46 – 57] | 67 [56 – 72] | 0.001 |
| CVP – mmHg | 5 [3 – 9] | 8 [5 – 11] | 0.02 |
| CI – L/min/m² | 2.34 [1.67 – 3.03] | 3.17 [2.39 – 4.03] | **< 0.001** |
| HR – beats/min | 115 [72 – 123] | 108 [81 – 117] | 0.31 |
| Lactate – mmol/L | 2.8 [2.0 – 4.95] | - | - |
| Norepinephrine |  |  |  |
| rate – µg/kg/min | 0.56 [0.28 – 1.43] | - | - |
| delay between initiation and CRT – hour | 12 [3 – 23] | - | - |
| CRT – s. | 5.68 [4.55 – 8.53] | 4.25 [3.24 – 5.38] | **< 0.001** |

*Fluid responsive patients were defined by an increase of CI ≥15% with volume expansion.*

*Values are presented as median [interquartile range]. p-values refer to the difference between before and after volume expansion.*

*CI: Cardiac index; CRT: Capillary refill time; CVP: Central venous pressure; DAP: Diastolic arterial pressure; HR: Heart rate; MAP: Mean arterial pressure; VE: Volume expansion.*

## **Supplementary table 2. Effect of volume expansion on hemodynamic variables in patients with baseline capillary refill time ≥3 seconds, depending on the changes in capillary refill time.**

| **Baseline CRT value ≥ 3 seconds and fluid responders patients**  **n = 13** | | | |
| --- | --- | --- | --- |
|  | **∆CRT significant**  **n = 8** | **∆CRT non-significant**  **n = 5** | **p-value** |
| ∆MAP – mmHg | 21 [6 – 43] | 56 [22 – 73] | 0.09 |
| ∆CI – L/min/m² | 24 [20 – 37] | 39 [23 – 78] | 0.28 |
| Arterial lactate – mmol/L | 2.9 [2.1 – 4.3] | 2.8 [1.7 – 5.7] | 0.97 |
| Norepinephrine |  |  |  |
| rate – µg/kg/min | 0.65 [0.31 – 1.37] | 0.43 [0.09 – 1.51] | 0.65 |
| delay between initiation and CRT – hour | 10 [2.0 – 19.2] | 24 [3.6 – 31.3] | 0.28 |

*CI: cardiac index; CRT: capillary refill time; ∆CI: changes in cardiac index; ∆CRT: changes in capillary refill time; ∆MAP: changes in mean arterial pressure.*

## **Supplementary table 3. Effects of increasing norepinephrine on hemodynamic variables depending on the change in mean arterial pressure****.**

| **Change in MAP ≥ 15%**  **n = 28** | | | |
| --- | --- | --- | --- |
|  | **Before** | **After** | **p-value** |
| MAP – mmHg | 64 [60 – 70] | 86 [80 – 103] | **<0.0001** |
| DAP – mmHg | 49 [44 – 54] | 62 [58 – 78] | **<0.0001** |
| CVP – mmHg | 9 [7 – 12] | 10 [8 – 14] | 0.08 |
| CI – L/min/m² | 2.98 [1.98 – 3.64] | 3.08 [2.40 – 4.09] | **0.0002** |
| HR – beats/min | 87 [71 – 96] | 87 [72 – 97] | 0.66 |
| Blood temperature - °C | 37.0 [36.0 – 37.3] | 36.9 [36.1 – 37.2] | **0.97** |
| Arterial lactate – mmol/L | 2.4 [1.9 – 3.47] | - | - |
| Norepinephrine |  |  |  |
| rate – µg/kg/min | 0.23 [0.08 – 0.45] | 0.41 [0.29 – 0.70] | **<0.0001** |
| delay between initiation and CRT – hour | 68 [15 – 245] | - | - |
| CRT – s. | 2.13 [1.55 – 4.74] | 2.04 [1.07 – 4.09] | **0.02** |
| **Change in MAP <15%**  **n = 8** | | | |
|  | **Before** | **After** | **p-value** |
| MAP – mmHg | 75 [70 – 89] | 84 [71 – 100] | **0.03** |
| DAP – mmHg | 61 [51 – 65] | 63 [55 – 70] | 0.13 |
| CVP – mmHg | 11 [6 – 14] | 11 [6 – 14] | 0.44 |
| CI – L/min/m² | 2.57 [2.17 – 3.81] | 2.67 [2.16 – 3.83] | 0.19 |
| HR – beats/min | 90 [73 – 105] | 83 [72 – 106] | 0.99 |
| Blood temperature - °C | 37.1 [36.2 – 37.5] | 36.9 [36.2 – 37.5] | 0.50 |
| Lactate – mmol/L | 1.8 [1.7 – 2.6] | - | - |
| Norepinephrine |  |  |  |
| rate – µg/kg/min | 0.42 [0.13 – 0.58] | 0.52 [0.35 – 0.77] | 0.008 |
| delay between initiation and CRT – hour | 40 [14 – 134] | - | - |
| CRT – s. | 3.72 [2.88 – 6.05] | 3.83 [2.60 – 5.36] | 0.31 |

*Values are presented as median [interquartile range]. P value refers to the difference between before and after the increase of norepinephrine infusion rate.*

*CI: Cardiac index; CRT: Capillary refill time; CVP: Central venous pressure; DAP: Diastolic arterial pressure; HR: Heart rate; MAP: Mean arterial pressure.*

## **Supplementary table 4. Effect of increasing norepinephrine on hemodynamic variables in baseline CRT <3 seconds patients (upper panel), baseline CRT ≥3seconds and change in MAP <15% (middle panel) and baseline CRT ≥3seconds and change in MAP ≥15% (lower panel).**

| **Baseline CRT <3 seconds**  **n = 19** | | | |
| --- | --- | --- | --- |
|  | **Before** | **After** | **p-value** |
| MAP – mmHg | 64 [60 – 71] | 87 [81 – 106] | **< 0.001** |
| DAP – mmHg | 49 [46 – 55] | 64 [59 – 79] | **< 0.001** |
| CVP – mmHg | 9 [6 – 12] | 10 [6 – 12] | 0.14 |
| CI – L/min/m² | 3.23 [1.98 – 4.19] | 3.68 [2.50 – 4.22] | **0.005** |
| HR – beats/min | 79 [65 – 94] | 86 [64 – 89] | 0.52 |
| Arterial lactate – mmol/L | 2.2 [1.8 – 2.9] | - | - |
| Norepinephrine |  |  |  |
| rate – µg/kg/min | 0.23 [0.06 – 0.45] | 0.39 [0.29 – 0.73] | **< 0.001** |
| delay between initiation and CRT – hour | 61 [19 – 248] | - | - |
| CRT – s. | 1.85 [1.33 – 1.97] | 1.40 [0.92 – 1.99] | 0.19 |
| **Baseline CRT ≥ 3 seconds and change in MAP <15%**  **n = 6** | | | |
|  | **Before** | **After** | **p-value** |
| MAP – mmHg | 75 [72 – 87] | 84 [74 – 94] | 0.09 |
| DAP – mmHg | 61 [55 – 64] | 63 [56 – 68] | 0.47 |
| CVP – mmHg | 12 [8 – 17] | 13 [9 – 17] | 0.50 |
| CI – L/min/m² | 2.57 [2.37 – 3.50] | 2.67 [2.19 – 3.61] | 0.56 |
| HR – beats/min | 90 [73 – 107] | 89 [74 – 118] | 0.31 |
| Lactate – mmol/L | 1.8 [1.7 – 2.42] | - | - |
| Norepinephrine |  |  |  |
| rate – µg/kg/min | 0.38 [0.09 – 0.80] | 0.52 [0.27 – 0.94] | **0.03** |
| delay between initiation and CRT – hour | 40 [24 – 89] | - | - |
| CRT – s. | 4.35 [3.62 – 6.76] | 4.10 [3.39 – 6.09] | 0.44 |
| **Baseline CRT ≥ 3 seconds and change in MAP ≥15%**  **n = 11** | | | |
|  | **Before** | **After** | **p-value** |
| MAP – mmHg | 65 [56 – 80] | 85 [79 – 96] | **0.001** |
| DAP – mmHg | 49 [42 – 59] | 60 [56 – 67] | **0.002** |
| CVP – mmHg | 10 [8 – 13] | 12 [8 – 14] | 0.36 |
| CI – L/min/m² | 2.40 [1.53 – 3.51] | 2.75 [1.83 – 3.90] | **0.002** |
| HR – beats/min | 88 [82 – 101] | 92 [79 – 96] | 0.57 |
| Lactate – mmol/L | 2.9 [1.9 – 4.4] | - | - |
| Norepinephrine |  |  |  |
| rate – µg/kg/min | 0.27 [0.12 – 0.50] | 0.45 [0.29 – 0.80] | **0.001** |
| delay between initiation and CRT – hour | 171 [9 – 259] | - | - |
| CRT – s. | 5.02 [3.79 – 6.11] | 4.99 [3.55 – 5.42] | 0.24 |

*Values are presented as median [interquartile range]. P-values refer to the difference between before and after the increase of norepinephrine infusion rate.*

*CI: Cardiac index; CRT: Capillary refill time; CVP: Central venous pressure; DAP: Diastolic arterial pressure; HR: Heart rate; MAP: Mean arterial pressure.*

## **Supplementary table 5. Univariate linear regression analysis for determination of the absolute value of capillary refill time.**

|  | **ß** | **95% CI** | **p-value** |
| --- | --- | --- | --- |
| **Baseline characteristics** |  |  |  |
| Age (years) | 0.39 | - 0.18 to 0.96 | 0.17 |
| Male sex | -0.05 | - 0.07 to – 0.02 | **0.0004** |
| BMI (kg/m²) | 0.008 | - 0.38 to 0.40 | 0.97 |
| SAPS II (points) | 1.832 | 1.017 to 2.647 | **< 0.0001** |
| SOFA (points) | 0.05 | - 0.34 to 0.45 | 0.79 |
| Pre-existing conditions |  |  |  |
| Coronary artery disease | 0.007 | - 0.01 to 0.03 | 0.45 |
| Chronic heart failure | 0.01 | - 0.002 to 0.03 | **0.08** |
| COPD | 0.003 | - 0.007 to 0.006 | 0.87 |
| Chronic kidney disease | 0.006 | - 0.009 to 0.02 | 0.46 |
| Diabetes | 0.009 | - 0.017 to 0.03 | 0.48 |
| Cirrhosis | - 0.002 | - 0.02 to 0.01 | 0.75 |
| Hypertension | 0.002 | - 0.02 to 0.03 | 0.86 |
| Source of infection |  |  |  |
| Lung | - 0.03 | - 0.05 to 0.0005 | **0.052** |
| Abdomen | 0.03 | 0.008 to 0.04 | **0.0047** |
| Urinary tract | - 0.002 | - 0.01 to 0.009 | 0.71 |
| Others | 0.0006 | - 0.02 to 0.02 | 0.95 |
| Mechanical ventilation at inclusion | - 0.03 | - 0.05 to - 0.02 | **0.0002** |
| PEEP (cmH_2_O) | - 0.2 | - 0.41 to 0.006 | **0.06** |
| C_RS_ (mL/cmH_2_O) | - 0.5 | - 1.36 to 0.36 | 0.25 |
| Respiratory rate (/min) | - 0.001 | - 0.24 to 0.24 | 0.99 |
| **Hemodynamic data** |  |  |  |
| Lactate (mmol/L) | 0.57 | 0.40 to 0.74 | **< 0.0001** |
| MAP (mmHg) | - 0.57 | - 1.45 to 0.3 | 0.19 |
| DAP (mmHg) | - 0.64 | - 1.29 to 0.005 | **0.052** |
| CVP (mmHg) | - 0.57 | - 0.26 to 0.30 | 0.88 |
| CI (L/min/m²) | 0.029 | - 0.11 to 0.007 | **0.09** |
| Heart rate (beats/min) | 1.49 | 0.27 to 2.71 | **0.02** |
| Body temperature (°C) | - 0.02 | - 0.08 to 0.04 | 0.5 |
| Norepinephrine |  |  |  |
| Doses (µg/kg/min) | 0.05 | 0.01 to 0.08 | **0.007** |
| Delay between initiation and intervention (hours) | - 4.713 | - 11 to 1.9 | 0.16 |
| **Outcome** |  |  |  |
| ICU mortality | 0.03 | 0.006 to 0.06 | **0.018** |

*BMI: Body mass index; CI: Cardiac index; COPD: Chronic obstructive pulmonary disease; C_RS_: Compliance of the respiratory system; CRT: Capillary refill time; CVP: Central venous pressure; DAP: Diastolic arterial pressure; HR: Heart rate; ICU: Intensive care unit; MAP: Mean arterial pressure; PEEP: Positive end-expiratory pressure; RR: Respiratory rate; SOFA: Sequential organ failure assessment; SAPS: Simplified acute physiology score.*

## **Supplementary table 6. Multivariate linear regression analysis to assess the determinants of the absolute value of capillary refill time.**

|  | **Model 1** | | | **Model 2** | | |
| --- | --- | --- | --- | --- | --- | --- |
| **Variables** | ß | 95 % CI | p-value | ß | 95 % CI | p-value |
| Lactate (per 1 mmol/L increment) | 0.70 | 0.49 to 0.91 | **< 0.0001** | 0.71 | 0.44 to 0.99 | **< 0.0001** |
| CI (per 1 L/min increment) | - 0.36 | - 0.98 to 0.25 | 0.24 | - 0.48 | - 1.14 to 0.19 | 0.16 |
| Heart rate (per 1 beat/min increment) |  |  |  | 0.02 | - 0.01 to 0.05 | 0.18 |
| DAP (per 1 mmHg increment) |  |  |  | 0.04 | - 0.09 to 0.06 | 0.72 |
| Norepinephrine dose (per 1µg/kg/min increment) |  |  |  | - 0.58 | - 1.76 to 0.59 | 0.33 |
| SAPS II (per 1 point increment) |  |  |  | 0.007 | - 0.04 to 0.06 | 0.77 |

*CI: Cardiac index; DAP: Diastolic arterial pressure, SAPS: Simplified acute physiology score.*

*Model 1 only included variables selected by step-by-step backward selection with removal of all items with a p-value >0.1: lactate and CI.*

*Model 2 selected all physiological variables of interest with a p value < 0.1 at univariate linear regression analysis (Supplementary table 2): lactate, CI, DAP, Norepinephrine dose and SAPS II.*

# **SUPPLEMENTARY FIGURES**

## **Supplementary figure 1. Least significant change (A) and precision (B) of capillary refill time measurement depending on the number of averaged measurements.**


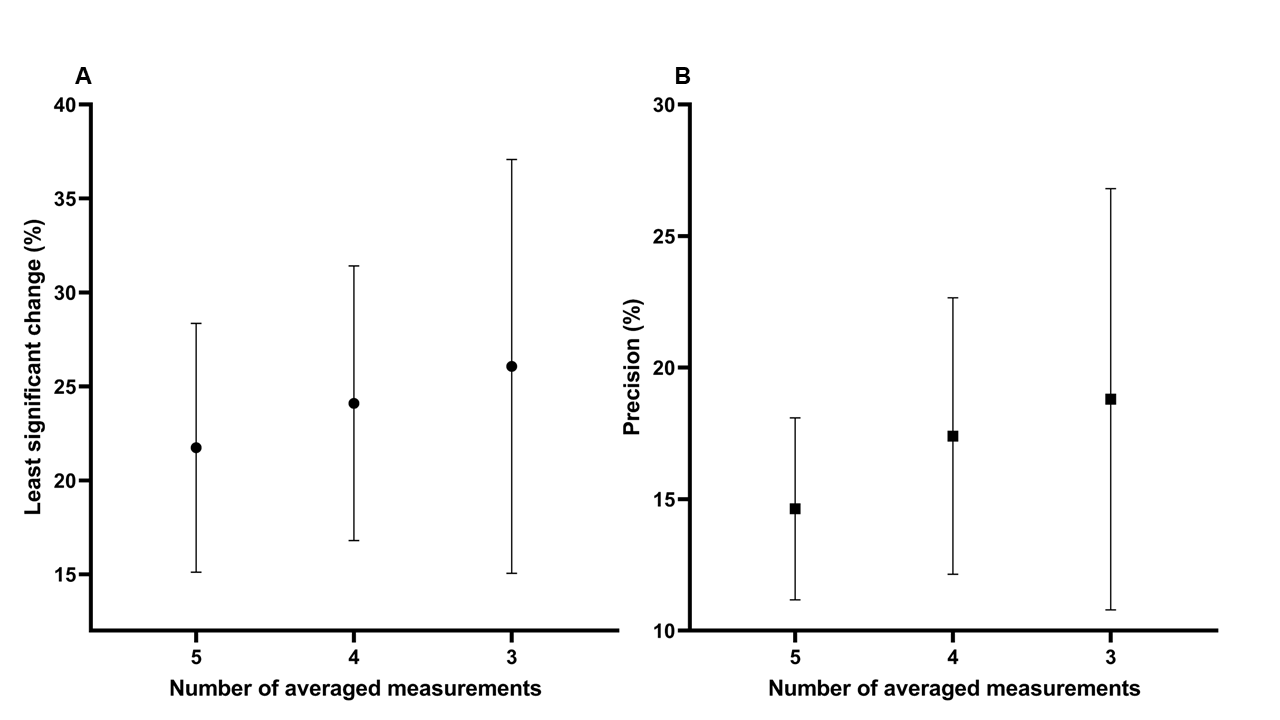


## **Supplementary figure 2. Correlation between capillary refill time and mean arterial pressure (A), cardiac index (B) and central venous pressure (C).**


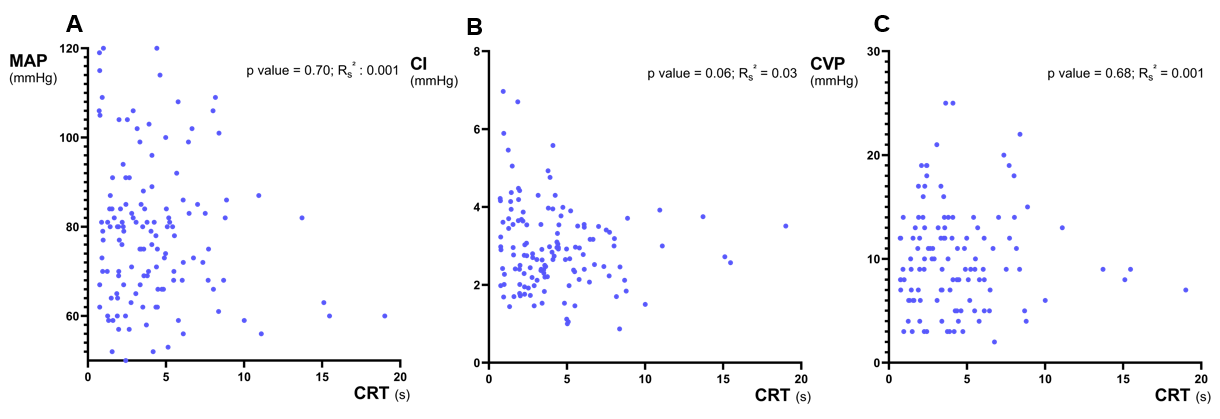


*CI: cardiac index; CVP: central venous pressure; MAP: mean arterial pressure.*

# **APPENDIX**

## **Appendix 1. STROBE Statement**

|  | Item No | Recommendation | Page No |
| --- | --- | --- | --- |
| **Title and abstract** | 1 | (*a*) Indicate the study’s design with a commonly used term in the title or the abstract | 1-3 |
|  |  | (*b*) Provide in the abstract an informative and balanced summary of what was done and what was found |  |
| Introduction | | | |
| Background/rationale | 2 | Explain the scientific background and rationale for the investigation being reported | 4 |
| Objectives | 3 | State specific objectives, including any prespecified hypotheses | 4 |
| Methods | | | |
| Study design | 4 | Present key elements of study design early in the paper | 5 and 7 |
| Setting | 5 | Describe the setting, locations, and relevant dates, including periods of recruitment, exposure, follow-up, and data collection | 5 |
| Participants | 6 | (*a*) Give the eligibility criteria, and the sources and methods of selection of participants. Describe methods of follow-up | 5 |
|  |  | (*b*) For matched studies, give matching criteria and number of exposed and unexposed |  |
| Variables | 7 | Clearly define all outcomes, exposures, predictors, potential confounders, and effect modifiers. Give diagnostic criteria, if applicable | 6-8 |
| Data sources/ measurement | 8* | For each variable of interest, give sources of data and details of methods of assessment (measurement). Describe comparability of assessment methods if there is more than one group | 7-8 |
| Bias | 9 | Describe any efforts to address potential sources of bias | 6-8 |
| Study size | 10 | Explain how the study size was arrived at | 8 |
| Quantitative variables | 11 | Explain how quantitative variables were handled in the analyses. If applicable, describe which groupings were chosen and why | 7-8 |
| Statistical methods | 12 | (*a*) Describe all statistical methods, including those used to control for confounding |  |
|  |  | (*b*) Describe any methods used to examine subgroups and interactions | 7-8 |
|  |  | (*c*) Explain how missing data were addressed |  |
|  |  | (*d*) If applicable, explain how loss to follow-up was addressed |  |
|  |  | (*e*) Describe any sensitivity analyses |  |
| Results | | |  |
| Participants | 13* | (a) Report numbers of individuals at each stage of study—eg numbers potentially eligible, examined for eligibility, confirmed eligible, included in the study, completing follow-up, and analysed | 9 and table 1 |
|  |  | (b) Give reasons for non-participation at each stage |  |
|  |  | (c) Consider use of a flow diagram |  |
| Descriptive data | 14* | (a) Give characteristics of study participants (eg demographic, clinical, social) and information on exposures and potential confounders | Table 1, page 9 |
|  |  | (b) Indicate number of participants with missing data for each variable of interest |  |
|  |  | (c) Summarise follow-up time (eg, average and total amount) |  |
| Outcome data | 15* | Report numbers of outcome events or summary measures over time | Table 2, Table 3 Supplementary table 1, 2, 3, and 4.;  Page 11 |

| Main results | 16 | (*a*) Give unadjusted estimates and, if applicable, confounder-adjusted estimates and their precision (eg, 95% confidence interval). Make clear which confounders were adjusted for and why they were included | 9-12  Figure 1 and Figure 2.  Table 2 and 3 |
| --- | --- | --- | --- |
|  |  | (*b*) Report category boundaries when continuous variables were categorized |  |
|  |  | (*c*) If relevant, consider translating estimates of relative risk into absolute risk for a meaningful time period |  |
| Other analyses | 17 | Report other analyses done—eg analyses of subgroups and interactions, and sensitivity analyses | Supplementary table 1 to 6. |
| Discussion | | | |
| Key results | 18 | Summarise key results with reference to study objectives | Page 13 - 16 |
| Limitations | 19 | Discuss limitations of the study, taking into account sources of potential bias or imprecision. Discuss both direction and magnitude of any potential bias | Page 16 |
| Interpretation | 20 | Give a cautious overall interpretation of results considering objectives, limitations, multiplicity of analyses, results from similar studies, and other relevant evidence | Page 13-16 |
| Generalisability | 21 | Discuss the generalisability (external validity) of the study results | Page 16 |
| Other information | | | |
| Funding | 22 | Give the source of funding and the role of the funders for the present study and, if applicable, for the original study on which the present article is based | NA |
